# A-calibration: assessment of prediction models for survival data under censoring

**DOI:** 10.1186/s12874-025-02671-6

**Published:** 2025-10-22

**Authors:** Mikkel Runason Simonsen, Rasmus Plenge Waagepetersen

**Affiliations:** 1 Department of Haematology, Clinical Cancer Research Unit, Aalborg University Hospital, Aalborg, 9000 Denmark; 2 Department of Mathematical Sciences, Aalborg University, Aalborg, 9000 Denmark

**Keywords:** Calibration, Predictive performance, Survival analysis, Random censoring, Goodness-of-fit testing, Probability integral transform

## Abstract

**Background:**

Evaluating the performance of predictive models for survival is essential before they can be trusted for real-world applications and decision making. While good measures such as the C-index are available for model discrimination, the toolbox for model calibration is much more limited in the time-to-event setting.

The method of D-calibration was therefore an important contribution that yields a single numeric value for calibration across the available follow-up time. D-calibration consists of performing a Pearson’s goodness-of-fit test on transformed survival times. Censored survival times are handled using an imputation approach which however tends to yield a conservative test and loss of power.

**Methods:**

In this paper, we introduce A-calibration based on Akritas’s goodness-of-fit test which is designed specifically for censored time-to-event data. Through theoretical arguments, simulations, and a case study, we compare A- and D-calibration as measures of calibration. In the simulation study, the power of each test to reject a false null-hypothesis was assessed for varying censoring mechanisms (memoryless, uniform and zero censoring), censoring rates, and parameter values of the predictive model considered.

**Results:**

The simulation study demonstrated that A-calibration had similar or superior power to D-calibration in all considered cases, and that D-calibration, unlike A-calibration, was particularly sensitive to censoring.

**Conclusions:**

Advantages of A-calibration compared to D-calibration have been demonstrated through theoretical considerations, a simulation study, and a case study, while no disadvantages relative to D-calibration were identified.

**Supplementary Information:**

The online version contains supplementary material available at 10.1186/s12874-025-02671-6.

## Background

Evaluating predictive performance of survival models is essential to validate the models for use in real-world settings. Predictive performance is usually measured in terms of *discrimination* and *calibration*. Discrimination is the degree to which the model is able to distinguish between high and low-risk cases, whereas calibration measures the accuracy of outcome predictions in comparison with the true outcome probabilities. The *integrated Brier score* (IBS) [1] and the *Concordance index* (C-index) [2] are commonly utilized performance measures. The Murphy decomposition of the Brier score shows that this score measures a combination of calibration, discrimination, and the probabilistic uncertainty of the outcome [3]. In contrast, the C-index is specifically a measure of discrimination. The IBS and the C-index can be estimated consistently using inverse probability of censoring weighting (IPCW) schemes [4, 5].

However, since the IBS and the C-index do not measure calibration specifically, calibration plots are often used for this purpose. These are visual tools rather than numerical quantities, making e.g. comparisons and optimization difficult. From calibration plots, certain summary statistics are sometimes extracted, including calibration intercept and slope. However, these quantities have been criticized with regards to interpretability [6] and have been characterized as *weak* measures of calibration, as they only measure average effects [7]. Therefore, other calibration measures have been developed based on the calibration curves, including the *estimated calibration index* [8]. Since calibration curves are not designed for time-to-event data, these calibration measures require a fixed time-point unlike the IBS and C-index which measure performance across the available follow-up.

Calibration has been described as the Achilles heel of predictive analytics, due to a combination of its great importance and the general lack of attention to calibration [9]. Recently, in the context of censored survival data, Haider et al. [10] introduced *D-calibration* that is not based on calibration curves and measures the calibration across the available follow-up. The main idea of D-calibration is to utilize the known distribution of event times transformed by the survival function, an idea previously used in the Cox-Snell residuals [11]. However, D-calibration uses an imputation approach to adjust for censoring that relies heavily on the null hypothesis considered. This can lead to considerable loss of power in the presence of censoring. In this paper we introduce a new method where we combine the transformation idea of D-calibration with a goodness of fit (GOF) test introduced by Akritas [12]. The new method which we coin A-calibration does not require data imputation under the null hypothesis. This can lead to greater power because dilution of data information due to imputation is avoided. The advantages of A-calibration are demonstrated by theoretical considerations, simulations, and a case study.

## Methods

### Notation

In this paper we consider independent and identically distributed (i.i.d.) randomly right-censored survival outcomes and predictors $(T_i,\Delta _i, Z_i)_{i=1}^n$ for $n\ge 1$ subjects. Here $Z_i$ is a vector of risk predictors for the *i*th subject, $X_i$ is the survival time with conditional survival function $S(\cdot \ | \ Z_i)$ given $Z_i$, $C_i$ is the censoring time, $T_i = \min (X_i,C_i)$ is the censored survival time, and $\Delta_i=1\lbrack X_i<C_i\rbrack$ is the censoring indicator (0 if censoring occurs and 1 otherwise). We also consider predictive models $\hat{S}$ of the survival functions, as well as observed survival outcomes and predictors ${\textbf {t}}=(t_i)_{i=1}^n$, $\varvec{\delta }=(\delta _{i})_{i=1}^n$, and ${\textbf {z}} = ({\textbf {z}}_i)_{i=1}^n$.

### Basic idea and assumptions

Throughout the paper, A- and D-calibration are presented and compared. The two approaches are very similar, as they both transform the observed survival data $({\textbf {t}}, \varvec{\delta }, {\textbf {z}})$ into a sample of known distribution under a null hypothesis, and then test whether the sample adheres to this distribution. For both methods, the null hypothesis more precisely means that the survival times are hypothesized to arise from a specific predictive model $\hat{S}$. For clarity, we emphasize that the null hypothesis is not a composite hypothesis that the true survival function belongs to some specific model class.

Assuming a continuous survival function, we employ the probability integral transform (PIT), $S(X_i \ | \ Z_i)$ for the survival times $X_i$, $i=1,\ldots ,n$. If *S* is strictly decreasing and thus possesses an inverse, it is trivial to show that the PIT transformed survival times follow the standard uniform distribution on [0, 1]. However, this also holds without strict monotonicity, which follows by utilizing the generalized inverse $S_{\text {gen}}^{-1}(y \ | \ z) = \inf \{t \ | \ S(t \ | \ z) \le y \}$. Thus, throughout the paper, a continuous but not necessarily strictly decreasing survival function is assumed.

Consider the uncensored *PIT residuals* $\hat{S}(x_i \ | \ {\textbf {z}}_i)$, $i=1,\ldots ,n,$ which approximately form a standard uniform sample if $\hat{S}$ is well calibrated. The central idea behind both A- and D-calibration is to test whether the PIT residuals adhere to the standard uniform distribution using goodness-of-fit (GOF) tests of the form

1 $$\begin{aligned} \sum \limits _{k=1}^K \frac{(N_k-E_k)^2}{E_k}, \end{aligned}$$

where $N_k$ and $E_k$ constitute observed and expected counts of PIT residuals (under the null hypothesis) in $I_k$, respectively, where $\{I_k\}_{k=1,\dots ,K}$ represents a partition of [0, 1] (or a subset thereof) into $K \ge 1$ intervals.

However, as the survival data is right-censored, the observed PIT residuals $\hat{S}(t_i \ | \ {\textbf {z}}_i)$ constitute a left-censored standard uniform sample under the null hypothesis.

The difference between A- and D-calibration lies in how the censoring of the PIT residuals is handled, which in turn influences how the terms $N_k$ and $E_k$ in (1) are defined as elaborated in the following sections.

Regarding the dependence between survival times and censoring times it is sufficient for both methods to assume conditional independence, that is $X \perp \!\!\!\!\perp C\ |\ Z$ where *X*, *C*, and *Z* is generic notation for a survival time, a censoring time and a predictor. This is because dependence between *X* and *C* through *Z* vanishes after the transformation to PIT residuals, as shown in Proposition 1.

#### Proposition 1

Let *X* be a survival time with survival function $S( \cdot \ | \ Z)$ depending on a predictor *Z* and let *C* be a right-censoring time such that $X \perp \!\!\!\!\perp C \ | \ Z$. Then the transformed survival and censoring times are independent, i.e. $S(X \ | \ Z) \perp \!\!\!\!\perp S(C \ | \ Z)$.

#### Proof

Let $$A,B \subseteq [0,1]$$. Then

$$\begin{aligned} P\left( S(X \ | \ Z) \in A, S(C \ | \ Z) \in B\right)= & \mathbb {E}\left[ P\left( S(X \ | \ Z) \in A, S(C \ | \ Z) \in B\right) \ | \ Z \right] \\= & \mathbb {E}\left[ P\left( S(X \ | \ Z) \in A \ | \ Z \right) P\left( S(C \ | \ Z) \in B \ | \ Z \right) \right] \\= & P\left( S(X \ | \ Z) \in A \right) \mathbb {E}\left[ P\left( S(C \ | \ Z) \in B \ | \ Z \right) \right] \\= & P\left( S(X \ | \ Z) \in A \right) P\left( S(C \ | \ Z) \in B \right) \end{aligned}$$

where the first and last equalities follow from the Law of Total Expectation, the second equality follows since $X \perp \!\!\!\!\perp C \ | \ Z$, and the third equality follows from the fact that the PIT residuals are independent of the predictors.

In the following two sections, the particularities of each of the methods are discussed.

### D-calibration

For D-calibration, in the case of no censoring, the idea would simply be to use a Pearson’s GOF test for standard uniformity, corresponding to a test statistic such as (1) with $N_k=\sum_{i=1}^n1\lbrack\widehat S(x_i\;\vert\;{\textbf{z}}_i)\in I_k\rbrack$ and $E_k = |I_k|$, i.e.

$$\chi^2=\sum_{k=1}^K\frac{\left(\left(\sum_{i=1}^n1\lbrack\widehat S(x_i\;\vert\;{\textbf{z}}_i)\in I_k\rbrack\right)-n\vert I_k\vert\right)^2}{n\vert I_k\vert},$$

As mentioned above, due to right-censoring, the PIT residuals form a left-censored sample, and hence $\sum_{i=1}^n1\lbrack\widehat S(x_i\;\vert\;{\textbf{z}}_i)\in I_k\rbrack$ is unknown. Assuming

$$\begin{aligned} \hat{S}(X_i \ | \ Z_i ) \ | \ (\Delta _i = 0, T_i = t_i,Z_i=z_i) \sim \text {unif}(0,\hat{S}(t_i \ | \ z_i)),i=1,\dots ,n, \end{aligned}$$

which follows under the null hypothesis due to Proposition 1, the contribution to the $k'th$ interval of the *i*’th subject censored at time $t_i$ is modified to ([10], Appendix B.5)

$$\begin{aligned} \left\{ \begin{array}{ll} 0 & \text {for } \hat{S}(t_i\ | \ {\textbf {z}}_i) \le \inf (I_k), \\ \frac{\hat{S}(t_i\ | \ {\textbf {z}}_i) - \inf (I_k)}{\hat{S}(t_i \ | \ {\textbf {z}}_i)} & \text {for } \hat{S}(t_i\ | \ {\textbf {z}}_i) \in I_k, \\ \frac{|I_k|}{\hat{S}(t_i\ | \ {\textbf {z}}_i)} & \text {for } \hat{S}(t_i\ | \ {\textbf {z}}_i) \ge \sup (I_k), \end{array}\right. \end{aligned}$$

where $\inf (I_k)$ and $\sup (I_k)$ represents the infimum and supremum of $I_k$. That is, for a subject censored at time $t_i$, the contribution is evenly distributed among the intervals in accordance with the interval lengths in $[0,\hat{S}(t_i \ | \ z_i))$ where the unobserved $\hat{S}(x_i \ | \ z_i)$ belongs. Using this approach to handle censoring, the expected proportion of PIT residuals within each interval equals the length of the intervals if $\hat{S} = S$, provided the true survival function is strictly decreasing ([10], Theorem 2B). A predictive model is considered D-calibrated if its associated *p*-value exceeds a specified significance level [10]. Furthermore, the performance of multiple predictive models can be compared on validation data by contrasting the test statistics.

The approach outlined above is in essence an imputation strategy, where the unknown $N_k, k =1,\dots ,K$ are imputed using the observed PIT residuals and the null hypothesis. This can make the imputed transformed sample appear close to a uniform sample despite possible differences between the true data distribution and the hypothesized one. As the GOF assesses the constrast between the observed $N_k$ and the expected $E_k$ under the null hypothesis, and $N_k$ itself is then imputed using the very same null hypothesis tested for, D-calibration can become a conservative test (low Type I error) under censoring at the cost of reduced power (greater Type II error). This issue was also identified by Haider et al. [10]. It was in particular highlighted how heavy zero censoring could be problematic. The impact of several different censoring schemes, including zero censoring, is considered in the simulation study (“Simulation study” and “Simulation study” sections).

### A-calibration

Given the discussion in the previous section, better handling of censoring is of key interest in connection with GOF testing. Our suggestion is to use Akritas’ Pearson-type GOF test introduced by Akritas [12] and developed specifically for randomly right-censored i.i.d. samples. Consider independent survival and censoring times $U_i$ and $V_i$ with distribution functions *F* and *G*, and let *H* denote the distribution function of $\min (U_i, V_i)$ for $i=1,\dots ,n$. The supports of *F* and *G* could be the entire positive real line or subsets thereof. The idea is to construct a test statistic on the form of (1) where $N_k=\sum_{i=1}^n1\lbrack u_i<v_i,u_i\in I_k\rbrack$ and $E_k$ are the observed and expected (under the null hypothesis) number of non-censored events occurring in $I_k$, and where the partitioning $\{I_k\}_{k=1,\dots ,K}$ is over the support of *H*, for the sample $(u_i,v_i)$, $i=1,\dots ,n$. The expected number of non-censored event-times in $I_k$ is

$$\begin{aligned} E_k = n\int _{I_k}(1-G)dF, \end{aligned}$$

which in particular depends on the censoring distribution *G*. To leave the censoring distribution unspecified and only assume random censoring, Akritas observes that $(1-G)(1-F)=1-H$ due to the independence of survival and censoring times, and proposes to estimate the censoring survival function as

$$\begin{aligned} 1-\hat{G} =\frac{(1-\hat{H})}{(1-F_0)}, \end{aligned}$$

where $F_0$ is the distribution function under the null-hypothesis and $\hat{H}$ is the empirical distribution function of the censored survival times. Inserting this estimator of the censoring distribution, the test statistic given in (1) follows a $\chi ^2$-distribution with *K* degrees of freedom under the null hypothesis.

We suggest using Akritas’ Pearsons-type GOF test on one minus the PIT residuals, $1-\hat{S}(t_1 \ | \ {\textbf {z}}_1), \dots , 1-\hat{S}(t_n \ | \ {\textbf {z}}_n)$, which constitutes a right-censored i.i.d. sample. With a slight abuse of wording, this sample will also be referred to as the PIT residuals. More precisely, we let $U_i=S(X_i \ | \ Z_i)$ and $V_i=S(C_i \ | \ Z_i)$ where $U_i$ and $V_i$ are independent by Proposition 1. Regarding the corresponding distribution functions, *F* has support [0, 1] while the common support of *G* and *H* is of the form [0, *a*] for $0 \le a \le 1$. Furthermore, under the null-hypothesis of $\hat{S}=S$, the sample follows a censored uniform distribution. Therefore, A-calibration is tested using (1), where

$$N_k=\sum_{i=1}^n1\lbrack\delta_i=1,1-\widehat S(t_i\;\vert\;{\textbf{z}}_i)\in I_k\rbrack\quad\text{and}\quad E_k=n\int_{I_k}\frac{1-\widehat H(t)}{1-t}\mathrm dt,$$

where $\hat{H}$ is the empirical distribution function of the PIT residuals.

Depending on the scientific context the upper limit *a* of the support of *H* may be known or unknown. If the censoring times $C_i$ are unbounded then $a=1$. If the censoring times are bounded, *a* may be less than one and we pragmatically choose $a=\sup \{t\in [0,1]: 1-\hat{H}(t)>0\}$, i.e. we use the empirical support of the transformed censored survival times. This modification is not covered by the theory in [12] but works well in the simulation studies. A-calibration thus proceeds as follows.

**Figure:**
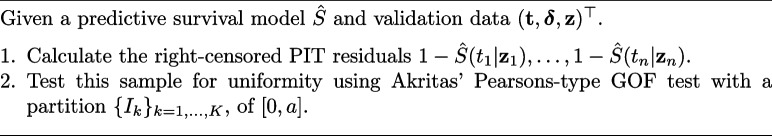
**Algorithm 1 **A-Calibration

Compared to D-calibration, A-calibration is advantageous because it avoids loss of power due to imputing data under the null hypothesis. Instead, the expected number of cases in each interval $I_k$ is adjusted to take censoring into account. Advantages of A-calibration compared to D-calibration are investigated in the subsequent simulation study.

### Simulation study

This simulation study compares the *power*, i.e. the probability of rejecting the null-hypothesis, of D-calibration and A-calibration in various circumstances. Throughout the study, a Weibull model is used as the true survival model, with survival function

$$\begin{aligned} S(t; \alpha , \sigma ) = \exp \left( -\left( \frac{t}{\sigma } \right) ^\alpha \right) \end{aligned}$$

for shape parameter $\alpha> 0$ and scale parameter $\sigma> 0$. The scale parameter depends on subject predictors and is given by $\sigma = \exp (\varvec{\beta }^\top {\textbf {z}}^*)$, where $\varvec{\beta }$ and ${\textbf {z}}^*$ are the vectors of true scale coefficients and subject-specific predictors.

Given predictors, the true survival model is used to simulate subject specific event-times, which are then right-censored according to one of three censoring schemes. The three censoring schemes considered are *memoryless*, *uniform* and *zero censoring*, where the censoring distribution *G* is either exponential, uniform with lower bound of zero, or dichotomous with outcomes 0 or $\infty$. For each scheme, parameters are chosen (rate, upper bound, or probability of zero) to achieve some specified censoring percentage *q* among the simulated subjects.

Simulated subjects form validation datasets used to evaluate calibration across different predictive models, all of which are misspecified Weibull models. Models are misspecified through missing predictors or misspecified shape or scale parameters. In the latter cases, misspecified are handled using a misspecification parameter $\lambda$, such that e.g. the scale is misspecified if scale coefficients $\lambda \varvec{\beta }$ are utilized for $\lambda \ne 1$. The simulation study is conducted as follows.

**Figure:**
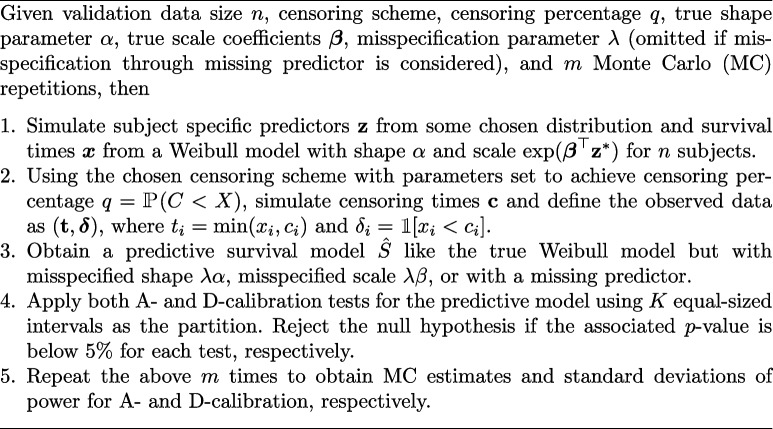
**Algorithm 2 **Simulation study

### Case study

In this case study the *Rotterdam* dataset from the R survival package [13] is considered. The Rotterdam data first described by Foekens et al. [14] concerns breast cancer patients registered in the Rotterdam tumor bank. The dataset consists of records for 2,982 patients of which 43% died during the available follow-up. Hence the censoring rate is 57%. The dataset contains information on the survival outcome, treatment received, year of surgery (year), age at surgery (age), and numerous clinical characteristics of the cancer and the patients.

In this case study we consider the predictors age, year, menopausal status, tumor size, cancer grade, number of involved lymph nodes, dosis received of progesterone and estrogen receptors, and whether the patients received hormones or chemotherapy. We perform repeated random splitting, dividing patients into a training cohort (70% of the data) and a validation cohort (30% of the data).

From the training cohort three predictive models are fitted: a simple Weibull regression considering only age as a predictor, a Weibull regression based on all predictors and a random survival forest (RSF) [15] based on all predictors. The minimal allowed node size for the RSF was treated as the tuning parameter and was selected over a grid of possible choices to minimize the out-of-bag (OOB) IBS. Finally, the predictive performance of all models was tested on the validation cohort using the C-index, the IBS, calibration plots, calibration intercept and calibration slope, as well as A- and D-calibration. These performance measures were averaged across the repeated training-test splits, and MC estimates along with standard errors were reported for all measures, except for the calibration plots, which were shown for a single training-test split only. The calibration plots, as well as the calibration intercept and slope, were evaluated at the 5-year follow-up mark. With regard to interpretation, C-index values closer to 1 indicate better discrimination; IBS values closer to 0 indicate better overall predictive performance; and calibration intercepts closer to 0 and slopes closer to 1 indicate better calibration. The R packages randomForestSRC [16] and pec [17] were used for training RSFs and evaluating predictive performance, respectively. All implementations, package usage, simulations, and the case study can be found in the supplementary R code.

## Results

### Simulation study

**Fig. 1:**
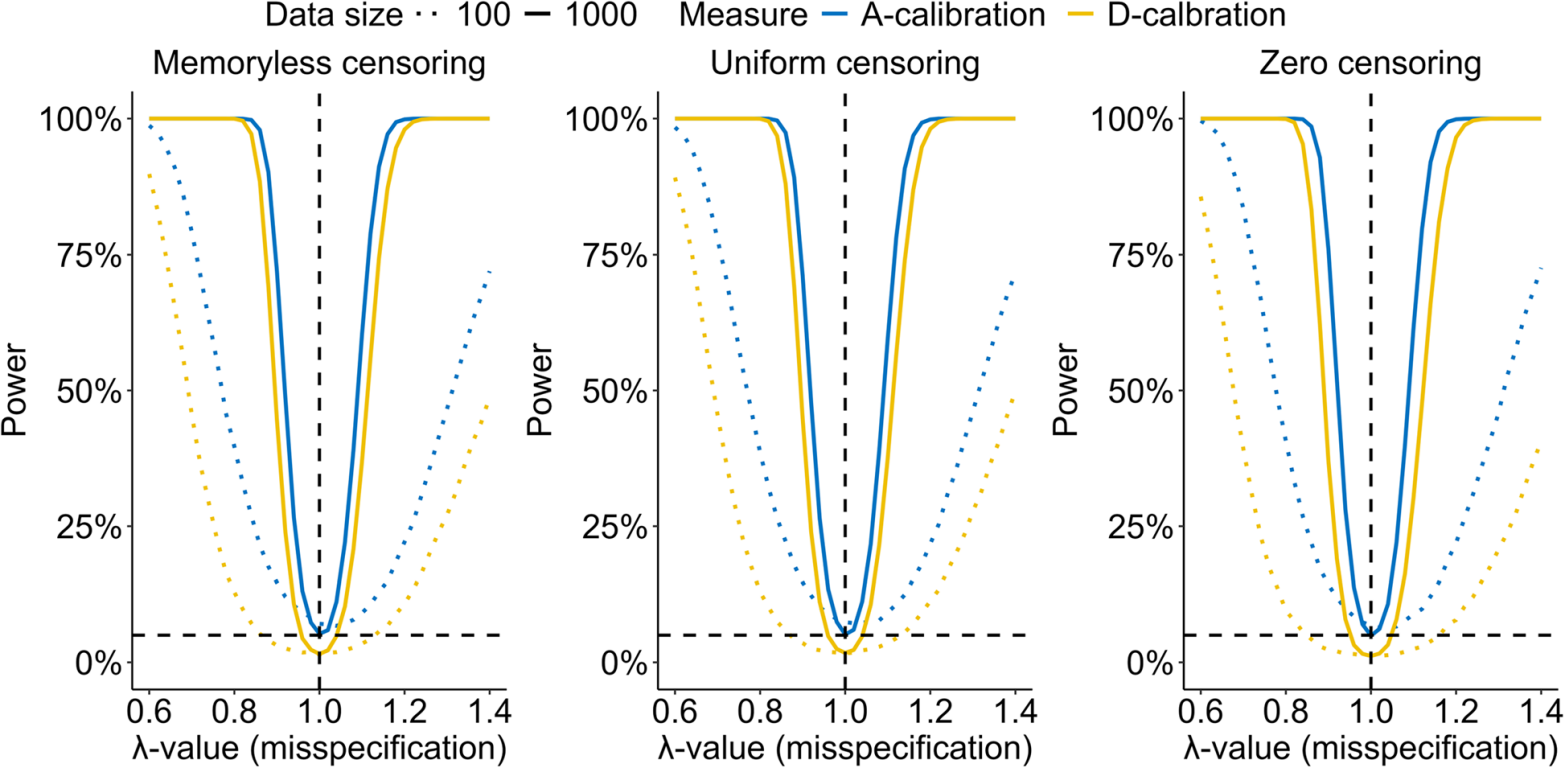
MC estimates of the power of A- and D-calibration with a censoring percentage of $q=20$ across varying $\lambda$-values controlling the misspecification of shape of the model, with $\lambda = 1$ yielding the true model. Estimates are based on 20,000 MC simulations for different validation data sizes and censoring schemes. The horizontal dashed line shows the 5% nominal significance level

The simulation study is conducted with $m = 20,000$ MC repetitions, true shape parameter $\alpha = 1$ (corresponding to an exponential model), and the scale coefficient vector is given by $\varvec{\beta } = \left(\log (2), \log \left(\frac{1}{2}\right), \log (2), \log (1.5)\right)$. Furthermore, the subject specific predictors are independently simulated as $z_1,z_2 \sim N(0,1)$ and $z_3, z_4\sim \text {Bernoulli(0.5)}$. The study varies the censoring scheme (memoryless, uniform, or zero censoring), censoring percentage *q* (0%−50%), misspecification parameter $\lambda$ (0.6-1.4) and validation data size *n* (100 and 1,000). The number of partitioning intervals, *K*, is set to 10, and evenly sized intervals are used. All MC standard errors for the power estimation were 0.1% or lower. For instance, if the power was estimated at 50% with an MC standard error of 0.1%, the corresponding 95% confidence interval would be [49.8%,50.2%]. As the number of MC repetitions was chosen to ensure that the MC standard errors are negligible compared to the observed differences between A- and D-calibration, we abstain from indicating MC standard errors in the simulation study plots. Figures 1 and S1 show the power of D-calibration and A-calibration for different $\lambda$ values, misspecifying the shape and scale, respectively, with a censoring percentage of $q = 20\%$.

**Fig. 2:**
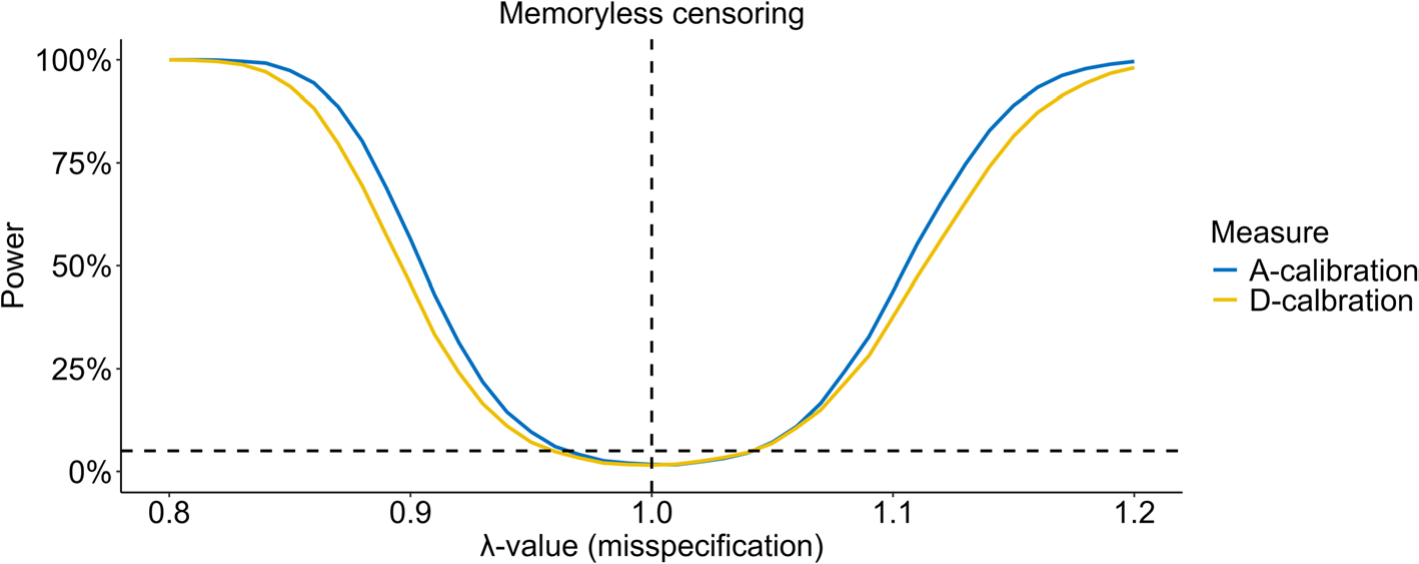
MC estimates of the power of A- and D-calibration with a censoring percentage of $q=20$ across varying $\lambda$-values controlling the misspecification of the shape of the model, with $\lambda = 1$ yielding the true model. Estimates are based on 20,000 MC simulations with validation data of size 1,000 and using the memoryless censoring scheme. In this simulation the significance level used for A-calibration was reduced to the nominal level of D-calibration at 1.3%

Across all conditions, regardless of misspecification, data size *n*, and censoring scheme, A-calibration always has a similar or superior power compared to D-calibration. For both tests, as the data size increases, the power curves get increasingly steep around the value $\lambda =1$, indicating that with sufficient validation data, either test can reliably reject a false null hypothesis. Although both tests use a nominal significance level of 5%, only the A-calibration power converges for increasing *n* towards the nominal level of 5% in the case of the true predictive model $\lambda =1$, while the power of D-calibration converges to a lower, censoring dependent value. For memoryless censoring with a 20% censoring rate, a data size of 1,000, and $\lambda =1$, D-calibration achieves a power, and hence an actual significance level, of 1.7% (Fig. 1). Figure 2 shows a similar plot, where the nominal significance level for A-calibration has been adjusted to the actual power of D-calibration (1.7%) under the null hypothesis. Even with this adjustment, A-calibration maintains a superior power throughout the range of $\lambda$ values misspecifying the shape.

**Fig. 3:**
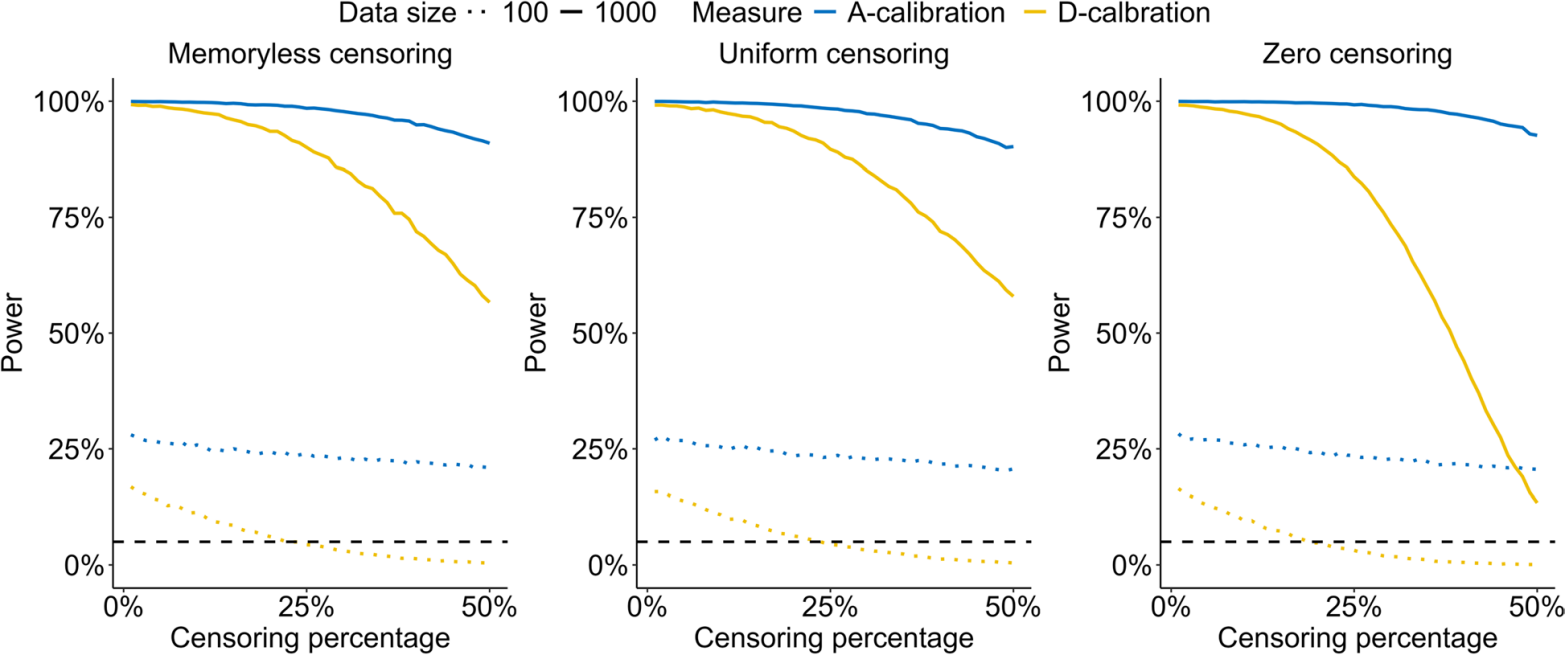
MC estimates of the power of A- and D-calibration with a misspecification of $\lambda = 0.85$ on the shape parameter of the model across varying censoring percentages *q*. Estimates are based on 20,000 MC simulations for different validation data sizes and censoring schemes

Figures 3, S2, and S3 illustrate that the reduced power of D-calibration is directly related to the censoring rate. Here we use misspecifications on shape, scale, or through missing predictor that correspond to models that are almost always rejected in the case of no censoring for $n=1,000$.

The power is reduced for both tests as the censoring percentage is increased but A-calibration is less affected by the increasing censoring percentage and maintains superior power, except for Fig. S2 where the power of D-calibration is slightly above that of A-calibration when $n=100$ and the censoring percentage is small. For larger data sizes, the censoring percentage has a diminishing impact on power. In particular, with a misspecification of $\lambda =0.8$ on the shape parameter and a datasize of 1,000, A-calibration maintains a power near 100% for all all censoring schemes, even when the censoring percentage reaches the extreme level of 50%. Finally, which censoring scheme had the greatest impact on power varied widely, depending on the misspecification of the model.

**Fig. 4:**
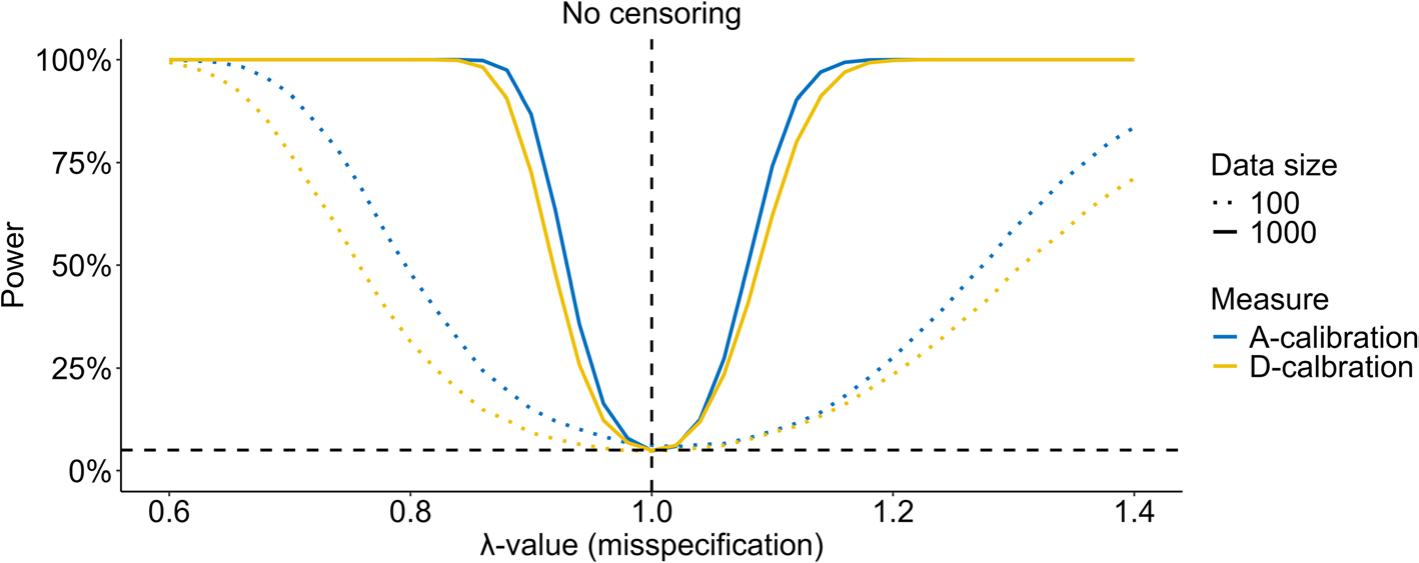
MC estimates of the power of A- and D-calibration with no censoring across varying $\lambda$-values controlling the misspecification of the shape of the model, with $\lambda = 1$ yielding the true model. Estimates are based on 20,000 MC simulations with validation data of size $n = 100$ and $n = 1,000$

Figures 4 and S4 compared A- and D-calibration in the case of no censoring. Notably, the actual Type I error for D-calibration here coincides with the nominal level in both figures. For Fig. 4, where the model’s shape was misspecified, A-calibration still maintained similar or superior power. For Fig. S4 however, where the model’s scale was misspecified, the powers of the methods were similar, with D-calibration occasionally exhibiting greater power.

### Case study

Considering the Rotterdam data, the average predictive performance of the three models trained on the training cohorts when predicting on the validation cohorts across 5,000 repeated training-test splits is found in Table 1. All MC standard errors are of order 0.001 or below, and thus, the uncertainty does not affect the conclusions drawn below.

Across all considered measures, the simple Weibull regression consistently performs the worst, while the random survival forest performs the best. However, while the other measures give us direct information regarding the quality of the predictions, A- and D-calibration only answer whether the given model could be the true model. Here, A-calibration would reject the simple Weibull model, whereas D-calibration would accept all of the considered models, even though the other performance measures clearly demonstrate the superior calibration (and model fit in general) of the random survival forest relative to the simple Weibull model.

**Table 1:** MC estimates of the predictive performance on the validation cohorts of the two Weibull regression models and the random survival forest trained on the training data through 5,000 repeated training-test splits

Measure	Simple Weibull	Weibull	Random survival forest
	Age	All predictors	All predictors
C-index	0.578	0.662	0.672
IBS	0.187	0.173	0.168
Intercept	−0.147	−0.134	0.002
Slope	0.592	1.106	1.086
A-calibration	0.0194	0.114	0.336
D-calibration	0.341	0.498	0.702

Furthermore, performances for a particular split (Table S1), including calibration plots for each of the models (Figs. S5-S7) show the same tendencies. However in this case, D-calibration resulted in a greater *p*-value for the simple Weibull model compared to the *p*-value for the full Weibull model. This violates the ranking of the models observed according to the other performance measures.

## Discussion

While the authors introducing D-calibration identified a weakness of the method in case of heavy zero censoring ([10], Appendix B.5), our simulation study has shown that other types of censoring can be problematic too, with the method becoming increasingly conservative depending on the censoring rate accompanied by loss of power. This emphasizes the need for improved calibration methodology.

Both A- and D-calibration are based on Pearson-type tests that can be written on the general form of Eq. (1). That is, both test statistics assumes a partitioning of [0, 1] into *K* intervals and constitute a sum of contrasts on each interval between what is observed $N_k$ and what is expected under the null hypothesis $E_k$, for $k=1,\dots ,K$. The primary difference between the two approaches is how censoring is incorporated into $N_k$ and $E_k$. For A-calibration, $N_k$ simply counts the number of non-censored (1-) PIT residuals belonging to the *k*th interval. The expected count is estimated using a non-parametric estimate of the censoring distribution based on the empirical distribution function of (1-) PIT residuals and the uniform distribution of the non-censored (1-) PIT residuals under the null hypothesis. For D-calibration, $E_k$ is given as *n* times the length of the *k*th interval which is the expected value under the null hypothesis ignoring censoring. However, this means that $N_k$ must be adjusted for censoring which is done using a kind of imputation leveraging the null hypothesis that is tested for. That is, the null hypothesis only influences $E_k$ for A-calibration, whereas it influences both $E_k$ and $N_k$ for D-calibration, and the degree to which $N_k$ is influenced depends on the censoring.

A-calibration offers several advantages. First, A-calibration has similar or superior power compared to D-calibration in all considered cases, and is notably less affected by censoring. The fact that A-calibration generally exhibited superior power in the absence of censoring is not particularly surprising. Although Akritas’ Pearson-type goodness-of-fit (GOF) test was primarily adopted for its ability to handle censoring, Akritas himself noted that in simulations without censoring, his test often outperformed Pearson’s traditional GOF test. This was likely due to the higher degrees of freedom in the test statistic [12]. Secondly, the actual significance level of A-calibration coincides with the nominal level as opposed to D-calibration where the true significance level depends greatly on censoring and is usually smaller than the nominal level. While Haider et al. [10] referred to the too frequent acceptance with D-calibration of the true model as “*p*-value boosting”, this may be considered unreliable because it depends on the censoring scheme and censoring rate.

A-calibration can be used to formally test if the suggested predictive model is the true model. However, in practice, the predictive model would never be the true underlying survival model, and hence even good models would have a risk of rejection greater than the chosen significance level. Therefore, if a binary accept/reject of the predictive model is demanded, it appears reasonable to choose a smaller significance level, for example, at 1%. Alternatively, the *p*-value can be interpreted as a calibration measure on a continuous scale, where higher *p*-values indicate better calibration. This also allows for comparison between multiple predictive models, where the model with the highest *p*-value would be considered to be the most well calibrated. However, one must be cautious with this approach, as comparing models based on the ranking of *p*-values requires that the validation datasets have the same size, follow the same distribution, and use the same partitioning. Therefore, using *p*-values, it is not straightforward to compare the performance of a new model to other models considered in previous studies. This is a clear limitation compared to other performance measures like the C-index and the IBS, which can be compared much more straightforwardly between models.

One issue of D-calibration which A-calibration has not solved is the arbitrary choice of partitioning intervals $\{I_k\}_{k=1,\dots ,K}$ that is left to the investigator to determine. While the impact of the choice of intervals can be investigated through a sensitivity analysis, it is still a disadvantage of the approach. Future research could involve applying a different GOF test to the PIT residuals which does not depend on such an partition, for instance by adapting Kolmogorov-Smirnov [18] or Anderson-Darling [19] tests to censored samples.

“Furthermore, a new issue has emerged with A-calibration that was not present with D-calibration. Specifically, in the case of bounded censoring, the censoring distribution *H* has support on the interval [0, *a*] for some $0 \le a \le 1$. We have proposed using the empirical support of the transformed censored survival times as a workaround, although this approach lacks theoretical justification. Despite this, in our simulations involving uniform censoring — even under heavy censoring scenarios where the upper support limit *a* is particularly small — A-calibration consistently outperformed D-calibration”.

An assumption underpinning the use of the probability integral transform is that the predictive survival model $\hat{S}$ is independent of the validation data. This means that the A-calibration of a model should be computed on a validation dataset independent of the data set used for training the predictive model.

## Conclusion

This paper introduces A-calibration as a new GOF testing method for predictive models in the context of censored survival data. Through theoretical considerations, a simulation study, and a case study, the method is shown to be superior to the existing alternative of D-calibration in terms of power under censoring.

## Supplementary Information

Supplementary Material 1.

## Data Availability

The code used to run the simulation studies and the case study, including accessing the Rotterdam dataset from the survival package in R, is included in a supportive file to the article.

## Abbreviations

IBS: Integrated brier score
C-index: Concordance index
IPCW: Inverse probability of censoring weighting
GOF: Goodness of fit
PIT: Probability integral transform
MC: Monte Carlo
RSF: Random survival forest
OOB: Out-of-bag

## References

[1] Graf E, Schmoor C, Sauerbrei W, Schumacher M. Assessment and comparison of prognostic classification schemes for survival data. Stat Med. 1999;18(17–18):2529–45. 10.1002/(SICI)1097-0258(19990915/30)18:17/18%3C2529::AID-SIM274%3E3.0.CO;2-5. 10474158 10.1002/(sici)1097-0258(19990915/30)18:17/18<2529::aid-sim274>3.0.co;2-5

[2] Harrell FE, Califf RM, Pryor DB, Lee KL, Rosati RA. Evaluating the yield of medical tests. J Am Med Assoc. 1982;247:2543–6. 7069920

[3] Murphy AH. A new vector partition of the probability score. J Appl Meteorol. 1973;12(4):595–600. 10.1175/1520-0450(1973)012%3C0595:ANVPOT%3E2.0.CO;2.

[4] Gerds TA, Schumacher M. Consistent estimation of the expected brier score in general survival models with right-censored event times. Biom J. 2006;48:1029–40. 10.1002/bimj.200610301. 17240660 10.1002/bimj.200610301

[5] Uno H, Cai T, Pencina MJ, D’Agostino RB, Wei LJ. On the C-statistics for evaluating overall adequacy of risk prediction procedures with censored survival data. Stat Med. 2011;30:1105–17. 10.1002/sim.4154. 21484848 10.1002/sim.4154 PMC3079915

[6] Stevens RJ, Poppe KK. Validation of clinical prediction models: what does the “calibration slope” really measure? J Clin Epidemiol. 2020;118:93–9. 10.1016/j.jclinepi.2019.09.016. 31605731 10.1016/j.jclinepi.2019.09.016

[7] Calster BV, Nieboer D, Vergouwe Y, Cock BD, Pencina MJ, Steyerberg EW. A calibration hierarchy for risk models was defined: from utopia to empirical data. J Clin Epidemiol. 2016;74:167–76. 10.1016/j.jclinepi.2015.12.005. 26772608 10.1016/j.jclinepi.2015.12.005

[8] Hoorde KV, Huffel SV, Timmerman D, Bourne T, Calster BV. A spline-based tool to assess and visualize the calibration of multiclass risk predictions. J Biomed Inform. 2015;54:283–93. 10.1016/j.jbi.2014.12.016. 25579635 10.1016/j.jbi.2014.12.016

[9] Calster BV, McLernon DJ, Smeden MV, Wynants L, Steyerberg EW, Bossuyt P, et al. Calibration: the Achilles heel of predictive analytics. BMC Med. 2019;17. 10.1186/s12916-019-1466-7. 10.1186/s12916-019-1466-7 PMC6912996 31842878

[10] Haider H, Hoehn B, Davis S, Greiner R. Effective ways to build and evaluate individual survival distributions. J Mach Learn Res. 2020;21:1–63.34305477

[11] Cox DR, Snell EJ. A General Definition of Residuals. Source J R Stat Soc Ser B Methodol. 1968;30:248–75. https://www.jstor.org/stable/2984505. Accessed 3 September 2025.

[12] Akritas MG. Pearson-Type Goodness-of-Fit Tests: The Univariate Case. Source J Am Stat Assoc. 1988;83:222–30.

[13] Therneau TM. A Package for Survival Analysis in R. 2024. R package version 3.8-3. https://CRAN.R-project.org/package=survival. Accessed 3 September 2025.

[14] Foekens JA, Peters HA, Look MP, Portengen H, Schmitt M, Kramer MD, et al. The Urokinase System of Plasminogen Activation and Prognosis in 2780 Breast Cancer Patients. Cancer Res. 2000;60(3):636–43. 10676647

[15] Ishwaran H, Kogalur UB, Blackstone EH, Lauer MS. Random survival forests. Ann. Appl Stat. 2008;2:841–60. 10.1214/08-AOAS169.

[16] Ishwaran H, Kogalur UB. Fast Unified Random Forests for Survival, Regression, and Classification (RF-SRC). Manual. 2025. R package version 3.4.1. https://cran.r-project.org/package=randomForestSRC. Accessed 3 September 2025.

[17] Gerds TA. pec: Prediction Error Curves for Risk Prediction Models in Survival Analysis. 2025. R package version 2025.06.24. 10.32614/CRAN.package.pec.

[18] Laha RG, Chakravarti JRIM. Handbook of Methods of Applied Statistics. John Wiley and Sons; 1967.

[19] Stephens MA. EDF statistics for goodness of fit and some comparisons. J Am Stat Assoc. 1974;69:730–7. 10.1080/01621459.1974.10480196.

