# Supplementary material for: A-calibration: assessment of prediction models for survival data under censoring
Source: BMC Med Res Methodol. 2025 Oct 22;25:236. doi: 10.1186/s12874-025-02671-6 (PMC12542389; doi:10.1186/s12874-025-02671-6)
Supplement: Supplementary file 1 — Supplementary Material 1. [file 12874_2025_2671_MOESM1_ESM.zip › Supplementary.pdf]

Supplementary

This document contains the supplementary figures and tables to the paper *A-Calibration: Assessment of Prediction Models for Survival Data under Censoring*

1 Supplementary Tables

**Measure**	**Simple Weibull** Age	**Weibull** All predictors	**Random Survival Forest** All predictors
C-index	0.582	0.685	0.707
IBS	0.189	0.167	0.160
Intercept	-0.205	-0.156	0.029
Slope	0.426	1.003	1.040
A-calibration	0.000	0.243	0.546
D-calibration	0.551	0.480	0.981

Table S1: Predictive performance on the validation cohort of the two Weibull regression models and the random survival forest for a single training-test split.

2 Supplementary Figures

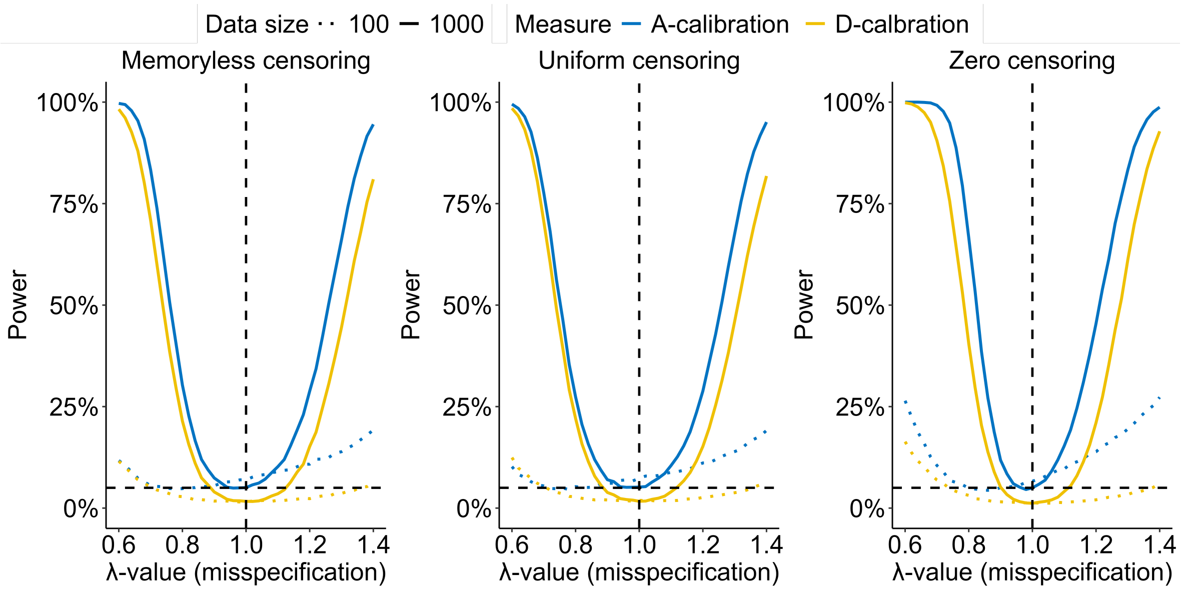

Figure S1: MC estimates of the power of A- and D-calibration with a censoring percentage of $q = 20$ across varying λ-values controlling the misspecification of the scale of the model, with $\lambda = 1$ yielding the true model. Estimates are based on 20,000 MC simulations for different validation data sizes and censoring schemes.

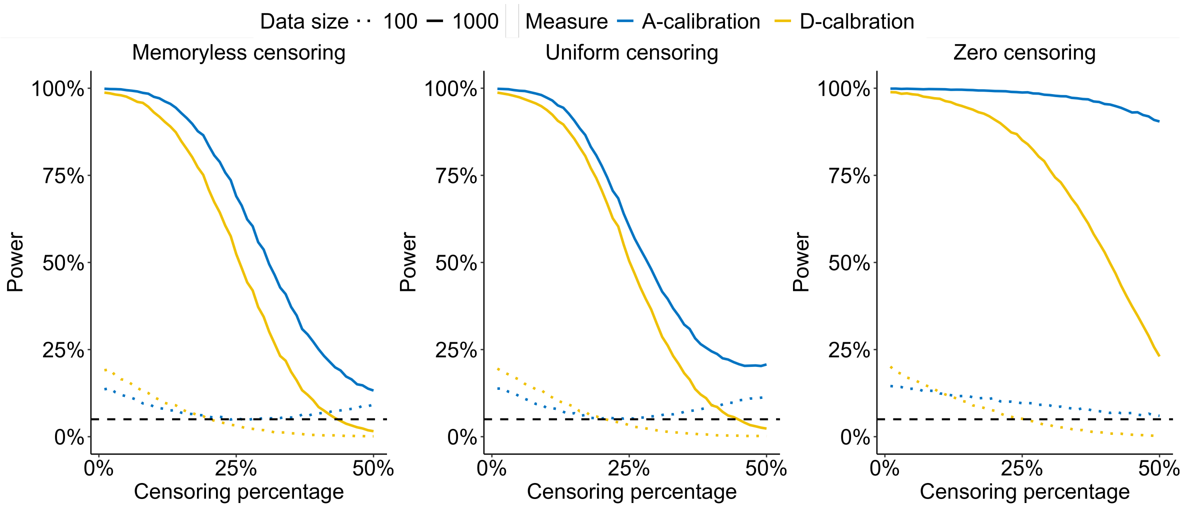

Figure S2: MC estimates of the power of A- and D-calibration with a misspecification of $\lambda = 0.7$ on the scale parameter of the model across varying censoring percentages q. Estimates are based on 20,000 MC simulations for different validation data sizes and censoring schemes.

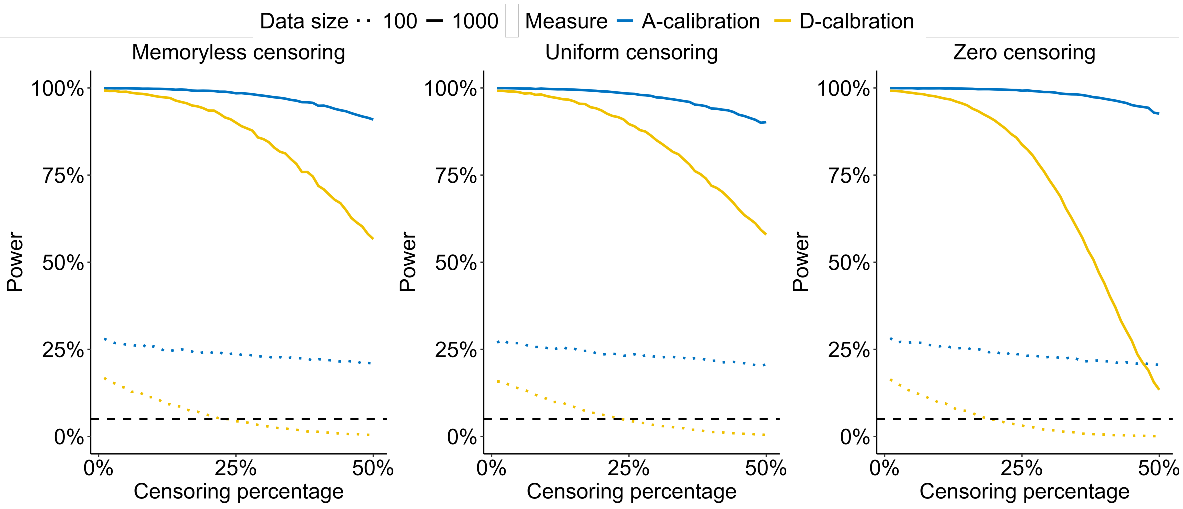

Figure S3: MC estimates of the power of A- and D-calibration with a misspecification of through a missing predictor across varying censoring percentages q. Estimates are based on 20,000 MC simulations for different validation data sizes and censoring schemes.

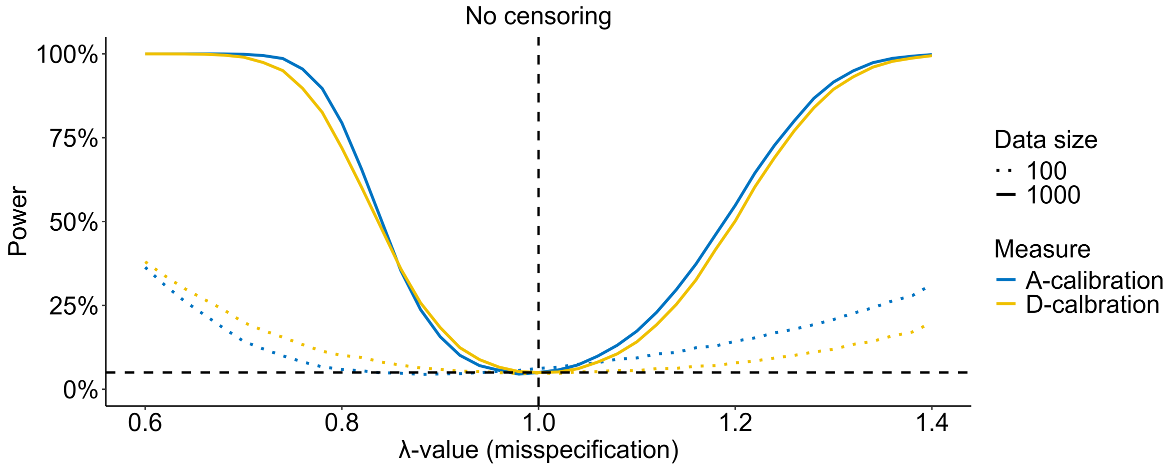

Figure S4: MC estimates of the power of A- and D-calibration with no censoring across varying λ-values controlling the misspecification of the shape of the model, with $\lambda = 1$ yielding the true model. Estimates are based on 20,000 MC simulations with validation data of size $n = 100$ and $n = 1,000$.

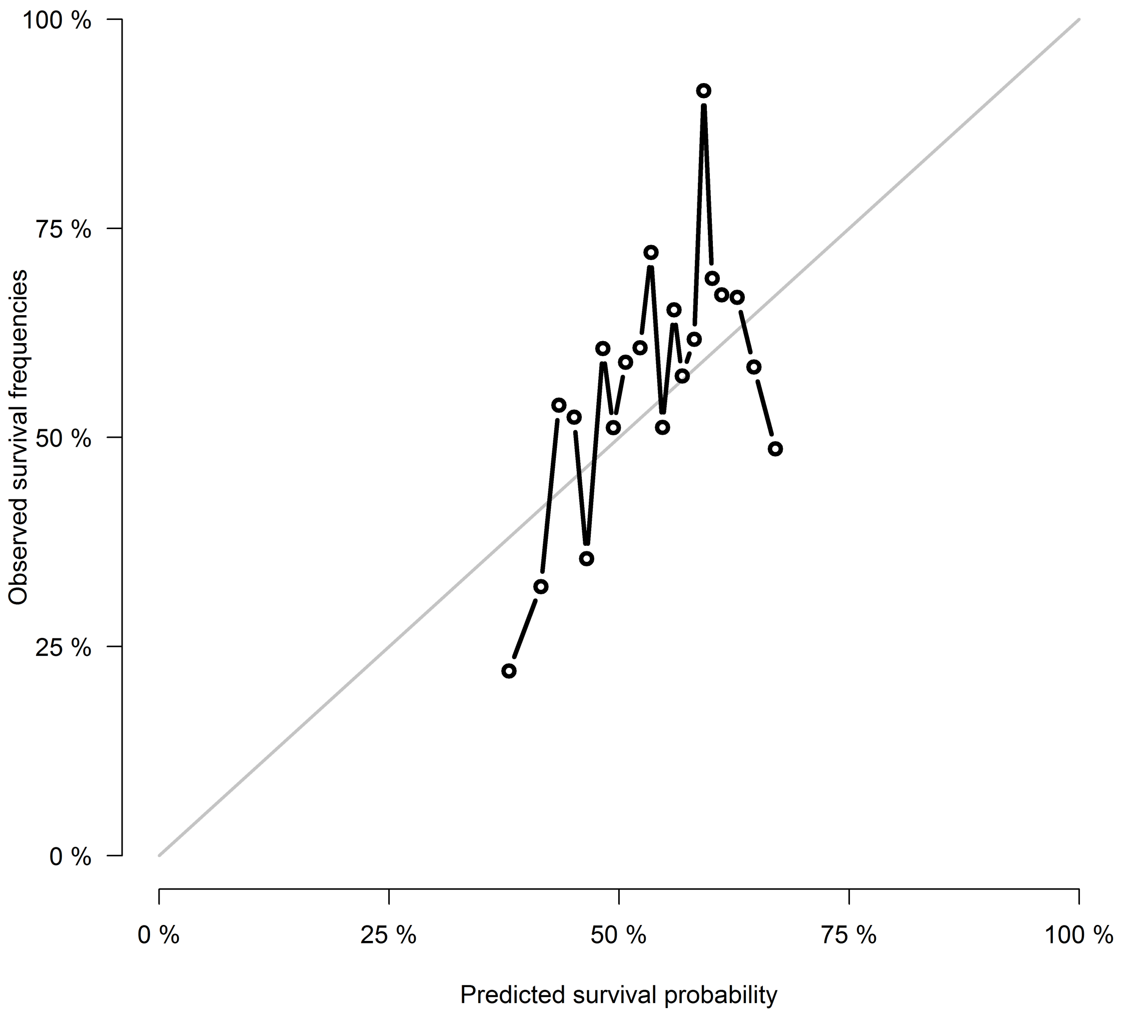

Figure S5: Calibration plot for a simple Weibull model using only age as predictor but on a single training-test split.

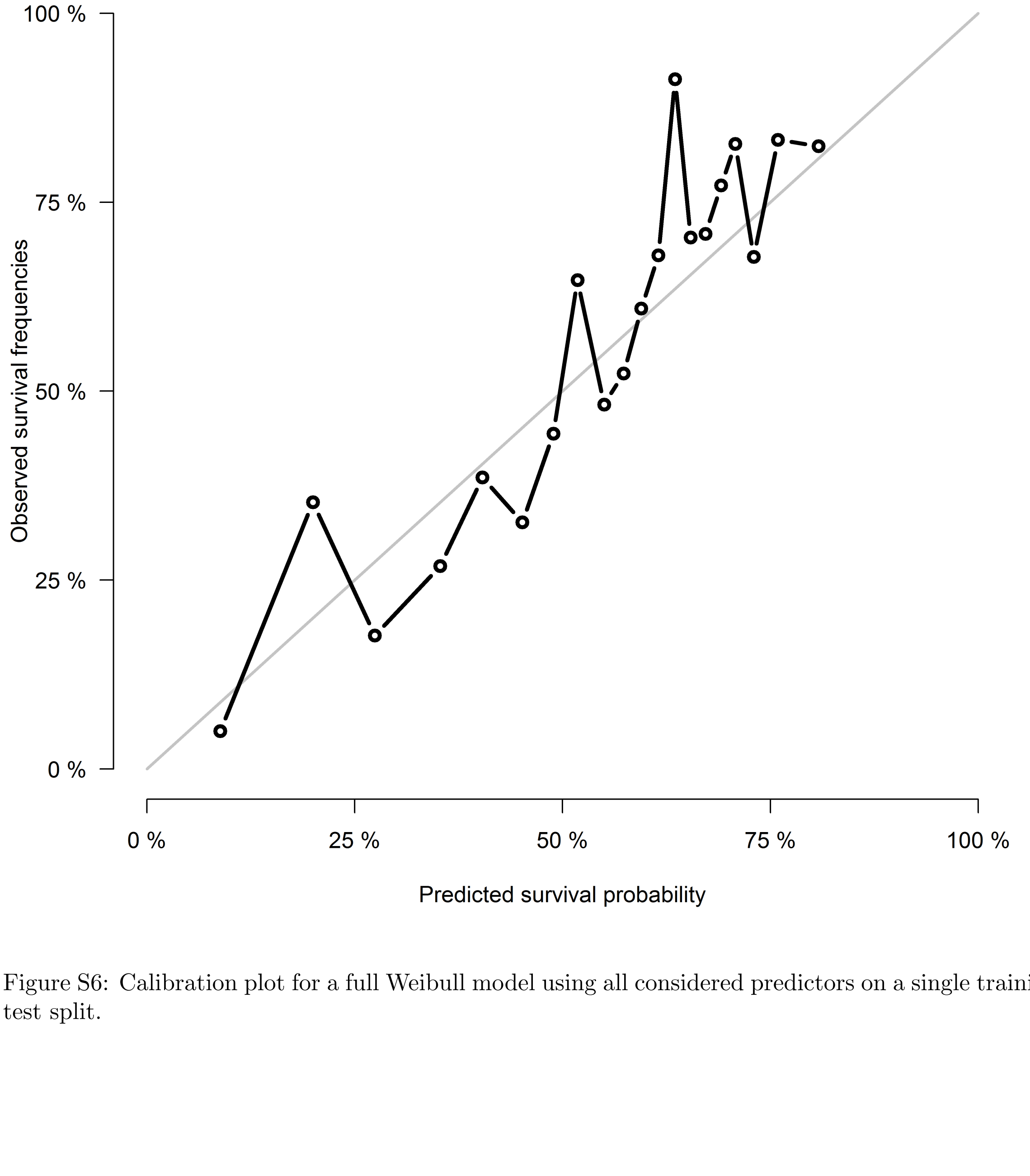

Figure S6: Calibration plot for a full Weibull model using all considered predictors on a single training-test split.

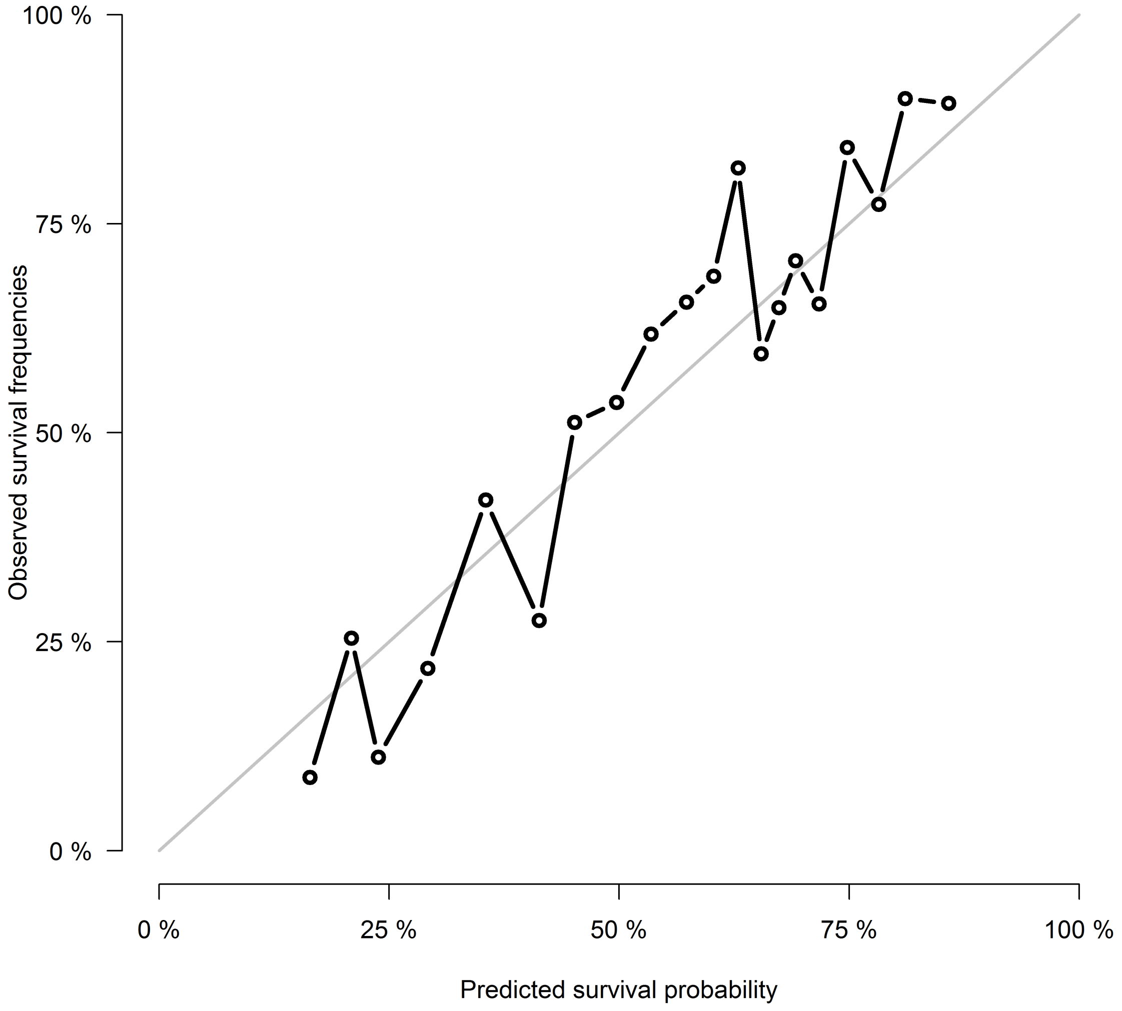

Figure S7: Calibration plot for trained random survival forest using all considered predictor for a single traning-test split.
